# PRevalence of Abuse and Intimate Partner Violence Surgical Evaluation (P.R.A.I.S.E.): rationale and design of a multi-center cross-sectional study. 

**DOI:** 10.1186/1471-2474-11-77

**Published:** 2010-04-23

**Authors:** PRAISE Investigators

**Affiliations:** 1 McMaster University, 293 Wellington Street North, Suite 110, Hamilton, Ontario L8L 8E7, Canada

## Abstract

**Background:**

Intimate partner violence (IPV) is described by the American Medical Association as "a pattern of coercive behaviors that may include repeated battering and injury, psychological abuse, sexual assault, progressive social isolation, deprivation, and intimidation." The long-term consequences of IPV include health risks, posttraumatic stress disorder, depression, and staggering economic costs for health care of victims. Intimate partner violence is often underreported among women who seek medical attention. The current study seeks to address the issue of possible underreporting of IPV in orthopaedic fracture clinics by establishing prevalence rates of IPV among women seeking treatment for musculoskeletal injuries.

**Methods/Design:**

We propose a cross-sectional multicenter study wherein 3,600 women will complete a self-reported written questionnaire across clinical sites in North America, Europe, and Australia. Recruitment of participants will take place at orthopaedic fracture clinics at each clinical site. The questionnaire will contain a validated set of questions used to screen for IPV, as well as questions that pertain to the participant's demographic, injury characteristics, and experiences with health care utilization. Female patients presenting to the orthopaedic fracture clinics will complete two validated self-reported written questionnaires (Woman Abuse Screening Tool (WAST) and the Partner Violence Screen (PVS)) to determine the prevalence of IPV in the past 12 months and in their lifetime. The two questionnaires were designed for rapid assessment of IPV status in emergency departments, family practice, and women's health clinics that we believe are similar to our intended setting of an orthopaedic clinic.

**Discussion:**

If the prevalence of IPV among women attending orthopaedic clinics is greater than the current perceptions of orthopaedic surgeons, this study will serve to advocate for the continued education of medical professionals to better recognize probable IPV cases and offer existing services to enhance the care of these patients.

## Background

Intimate partner violence (IPV) is described by the American Medical Association as "a pattern of coercive behaviors that may include repeated battering and injury, psychological abuse, sexual assault, progressive social isolation, deprivation, and intimidation" [1]. The long-term consequences of IPV include health risks, posttraumatic stress disorder, depression, and staggering economic costs for health care of victims [2].

The cumulative lifetime prevalence of domestic violence for women admitted to the Emergency Department is approximately 54 percent [2]. However, IPV is underreported among women who seek medical attention [2-4]. The American College of Surgeons position statement on IPV states that surgeons have the responsibility to identify IPV and appropriately treat women at risk of further harm [5]. In July 2009, the Canadian Orthopaedic Association (COA) released a position on the role of the orthopaedic surgeon in the care of abused women. The statement affirms that the "COA recognizes that IPV is a significant social determinant of morbidity and mortality, and that orthopaedic surgeons are well positioned to identify patients living with IPV and initiate an intervention. Therefore, the COA encourages its members to educate themselves further about IPV and considers it good medical practice to take steps to identify and offer assistance to its victims" [6]. In accordance with this position statement, the COA is working towards raising awareness of domestic violence as well as educating orthopaedic surgeons over the next year. This shows the timeliness of the issue in orthopaedic surgery.

Despite these positive initiatives, there is currently no data in orthopaedic literature to support the hypothesis that the prevalence of IPV in orthopaedic trauma clinics warrants additional resources to identify and manage victims [7,8]. The current study seeks to address the concern that IPV is underreported in orthopaedic fracture clinics by establishing prevalence rates of IPV among women seeking treatment for musculoskeletal injuries across multiple centers in different jurisdictions. This will be the first multi-center study to evaluate this critical issue in the field of orthopaedic trauma.

The proportion of women in Canada 15 years of age and older who have experienced physical or sexual violence in a marital or common-law union in the last 5 years is between 6 percent and 8 percent, affecting approximately 300,000 women in the province of Ontario [9]. This is likely an underreporting of the true rate since detection is hindered by the reluctance of respondents to talk openly about their IPV experience [10]. In documented cases of IPV in Canada, 25 percent of women reported that they were beaten, 20 percent reported choking, and 20 percent were sexually assaulted. Of the documented cases, 40 percent of women in Canada who have experienced IPV suffer a physical injury, and 15 percent of these cases are serious enough to warrant medical attention [10].

Several studies have found that IPV is underreported among women who seek medical attention [2-4]. Dearwater et al. noted that women who were treated at 11 community hospital emergency departments secondary to IPV-related acute trauma were identified in only 44 percent of all cases presented [3]. Davis et al. reported that of the victims who presented to a level I trauma center because of confirmed or probable IPV, only 26 percent received referrals to social services and 63 percent were discharged without any investigation into their safety at home [11]. The investigators further note that the number of IPV victims was underestimated because of the propensity of conflicting information in the patients' history, lack of information to adequately rule out IPV, and failing to actively screen for IPV among all cases who presented to their trauma service. Additionally, a recent study found that sprains, dislocations, fractures, and foot injuries accounted for 28 percent of all clinical manifestations of IPV among women who were identified in a 2-year period by the Minnesota Domestic Abuse Program [12]. Despite these findings, IPV remains underemphasized in this medical field [7].

The majority of orthopaedic surgeons (87%), however, in a Canada-wide survey believed that female victims of IPV accounted for less than 1 percent of patients in their care [7]. The findings of this survey suggested a misperception between surgeons' beliefs about the prevalence of IPV in their fracture clinics and reported rates of IPV in the community [7]. Current guidelines suggest that orthopaedic surgeons should play an active role in the identification of IPV victims and their timely referral to local agencies [7,13,14].

To explore prevalence rates of IPV in the fracture clinic setting, we conducted a pilot screening study of injured women across two trauma centers in Ontario. We found that one third of women have been victims of IPV (including physical, emotional, and sexual abuse) in the past 12 months. Furthermore, we found that 2.5 percent of women presented with injuries directly resulting from IPV [15]. While our findings were compelling, the generalizability between two level I trauma centers remained unknown. The current proposal aims to expand the generalizability and significantly increase the sample size to provide more precise estimates of IPV in the fracture clinic setting.

### Research Objectives and Hypotheses

The primary objective of this observational study is to determine the proportion of women who have experienced IPV in the past 12-months among women who present to orthopaedic fracture clinics for treatment of orthopaedic injuries. We define IPV as physical, emotional, or sexual abuse that is caused by an intimate partner such as a victim's spouse, common-law partner, or dating partner.

Secondary objectives include determining the proportion of women presenting to orthopaedic fracture clinics for the treatment of orthopaedic injuries who have experienced IPV in their lifetime; determining the proportion of women who present to orthopaedic clinics for treatment of orthopaedic injuries that present with an orthopaedic injury that was the direct result from IPV from a current and ongoing relationship; and investigating patients' previous experiences, knowledge, and perceptions with regards to approaching healthcare professionals about IPV.

We hypothesize that the prevalence of IPV among women who present to orthopaedic fracture clinics for treatment of orthopaedic injuries is sufficiently high to warrant targeted programs to assist orthopaedic surgeons in the clinic with identification and appropriate referral to specialists.

## Methods/Design

**Figure 1:**
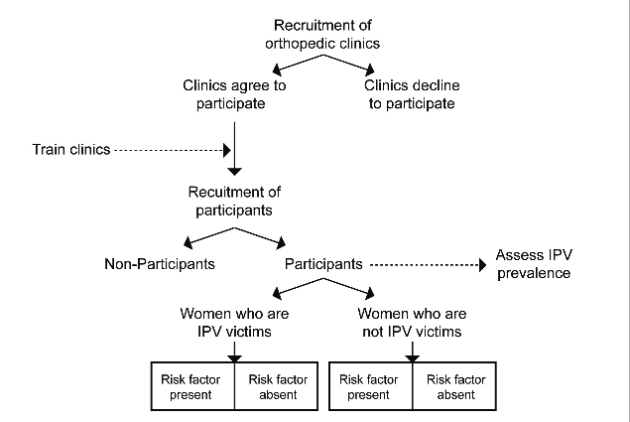
**Study Flow diagram**.

We propose a cross-sectional multi-center study wherein 3,600 women will complete a validated self-reported written questionnaire across ten clinical sites in North America, Europe, and Australia. Recruitment of participants will take place at the fracture clinic at each clinical site. The questionnaires will contain a validated set of questions used to screen for IPV, as well as questions that pertain to the participant's demographic, fracture characteristics, and experiences with health care utilization. A flow diagram of the study is presented in Figure 1.

### Rationale for an Observational Study Design

We explored the possibility of addressing our research questions by other less costly or less time-consuming study designs. Other methods considered were a retrospective cohort and a cross-sectional study using a self-reported written questionnaire sent by mail. The advantages and disadvantages of these study designs when compared to the proposed design are discussed below.

A retrospective cohort study would be less costly and time consuming than the prospective cohort study or case-control study proposed. A medical chart review can be undertaken at each participating clinic, where data collectors can review the charts for reported cases of IPV among patients who visited the clinic within a certain time period. Such a study would have a greater likelihood of undergoing an expedited ethics review than a study that requires active participation from the respondent. However, given that IPV is not properly recognized among health professionals [3,4,16], such a review would likely be unable to detect a significant proportion of IPV victims. Moreover, the non-standardization of the definition of IPV may also affect the internal validity of the study and result in an over reporting or underreporting of cases of IPV.

The use of a mailed questionnaire that can be completed and sent back by the participant is a potential strategy that would save costs over a study that requires the respondent to be present at the clinic to be enrolled. Female patients over the age of 18 years can be identified from the registries of the orthopaedic fracture and injury clinics and be mailed a written questionnaire and information letter about the study. Informed consent would be implied if the questionnaire is sent back to the study investigators. However, while such a study would be easier to implement than a prospective study, the administration of a mailed questionnaire has been met with limited success. In their study of abuse prevalence among patients visiting gynaecology clinics, Wijma et al. noted response rates as low as 67 percent among recruiting clinics that mailed questionnaires to eligible participants [4]. Campbell et al., in their attempt to assess the physical consequences of IPV with a questionnaire that was mailed, reported a response rate of only 12 percent [17].

In contrast to the use of mailed questionnaires, written self-reported surveys such as the questionnaire suggested in the proposed study design may have less likelihood of response bias. Use of such questionnaires has resulted in response rates between 62 percent and 95 percent among women who are invited into the study personally by research personnel and then administered the questionnaire if they chose to participate [1,4,18,19].

### Inclusion and Exclusion Criteria

All women who present to a recruiting orthopaedic or trauma clinic will be screened for eligibility. Our inclusion criteria for this observational study are: 1) The patient presents to the fracture clinic for her own appointment; 2) The patient is 16 or 18 years of age or older; 3) The patient is able to read and write well enough to complete the study questionnaires; 4) The patient is being seen at the fracture clinic for the treatment of an orthopaedic injury; and 5) The patient is able to separate herself from anyone who accompanied her to the fracture clinic to complete the questionnaire in privacy. Clinical sites may include patients who are over the age of 16 years in the study if it is permitted by their Research Ethics Board. Some sites may not allow patients under the age of 18 to participate. Lowering the age requirement to 16, where possible, will improve the generalizability of the study and will allow us to determine the rates of IPV in a younger demographic.

The exclusion criteria for this study are: 1) The patient is considered too ill or injured to participate in the study; and 2) The patient is cognitively impaired and unable to participate in the study.

Similar inclusion and exclusion criteria have been used in other studies that sought women's experience with IPV [1,3,8,16]. Once a patient is deemed eligible, she will be invited to participate in the study by a female study coordinator. We will track the number of patients screened and their reasons for ineligibility.

### Primary and Secondary Outcome Measures

To measure the prevalence of IPV, our questionnaire will ask patients if their intimate partner had abused them physically, emotionally, or sexually in the past 12 months and throughout their lifetime. We believe that it is important that our study attempt to quantify levels of emotional and sexual abuse among our intended study sample because the various forms of IPV - physical, emotional, and sexual abuse - are concomitant with each other, and orthopaedic surgeons may serve as a second line of detection of IPV if victims are undetected in healthcare settings antecedent to their presentation to the orthopaedic clinic, such as the emergency department. Moreover, we take the stance that orthopaedic surgeons should be concerned about the holistic care of the individual instead of only being concerned with what is immediately treatable in their area of expertise. Therefore, to estimate the overall prevalence of IPV, we will combine the positive answers to the questions on physical, emotional, or sexual abuse.

We will also ask participants to complete two validated questionnaires that were designed for rapid assessment of IPV status in emergency departments, family practice, and women's health clinics that may be similar to our intended setting of an orthopaedic fracture clinic. We selected the Woman Abuse Screening Tool (WAST) and the Partner Violence Screen (PVS) for their psychometric properties, reliability, and specificity in identifying partner abuse. Moreover, a study that was published in 2006 reported that 94 percent of women who were administered both the PVS and WAST concurrently, as written questionnaires, considered them "easy" to complete [18]. Both questionnaires are widely used in IPV screening studies [18,20-26].

We chose to request that both questionnaires be administered to each participant to ensure that we are identifying all probable cases of IPV among women who attend orthopaedic clinics. In addition to the elucidation of physical abuse, the WAST contains questions that assess the levels of emotional and sexual abuse that we also feel are important to ascertain in our study, as mentioned previously.

Another research question that will be addressed in the study is what are patients' previous experiences and perceptions about discussing IPV with health care professionals. To that end, the questionnaire will also query the participant about her age, income, education, race/ethnicity, marital status, sexual orientation, and length of relationship. Additionally, participants will be queried about perceptions and previous experiences with reporting IPV in health care settings.

The questionnaire will also ask participants to record the characteristics of the injury that they are being seen for in the fracture clinic including: type of injury, how the injury occurred, locations of injury, and date of injury.

### Data Collection

The self-report component of the data collection will involve a written self-completed questionnaire. This method of data collection has been shown in a randomized trial to provide the least amount of missing data by the respondent and is generally favored over the use of a face-to-face interview or computer-based self-completed questionnaire [18].

**Figure 2:**
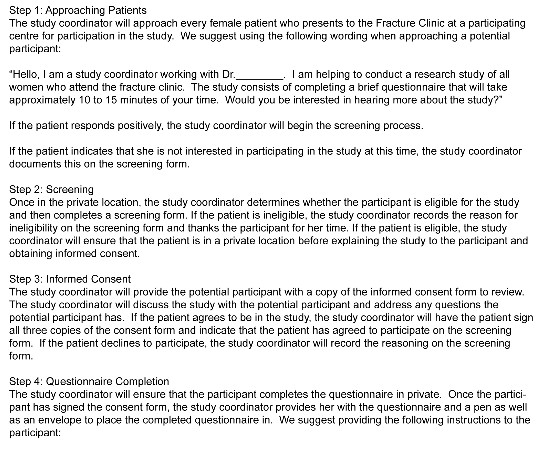
**Participant Enrollment Procedures**.

A female study coordinator at each participating clinical site will approach all female patients who present to the fracture clinic for participation in this study. Due to the sensitive nature of the study, the informed consent process and completion of the questionnaire will take place in a private location by the female study coordinator. A screening form will be completed for all patients that will document their eligibility and whether they agree to participate in this study. After providing informed consent, the female study coordinator will provide the participant with the questionnaire to complete. When the participant has completed the questionnaire, she will place it in a sealed envelope and return it to the study coordinator. This process is described in Figure 2.

### Ethical Considerations

Due to the nature of the research topic, care must be exercised when recruiting individuals to participate in the study. Women are often afraid of disclosing that they are a victim of IPV for fear of retaliation from the offender, stigmatization by the individuals that she discloses to, embarrassment, and police involvement [27]. Because of this fear, key ethical issues addressed in this study are: 1) requirement for free and informed consent; 2) respect for privacy and confidentiality; 3) respect for justice and inclusiveness; 4) minimizing harm; and 5) maximizing benefit. Each of these items are discussed in greater detail below.

If an individual agrees to be approached by a study coordinator, the study coordinator will first ascertain whether the individual is eligible for participation and ask the participant if she would be able to accompany the study coordinator by herself to a private location to participate in a women's health survey. At no point during this initial contact will there be any mention of the words "abuse" or "violence." If the potential participant is able to come by herself into the private location, the informed consent process will be carried out, and the study coordinator will remain in the private location to ensure that the participant is not interrupted as she completes the questionnaire.

To maximize the opportunity for free and informed consent while respecting privacy and confidentiality, the informed consent process will only take place privately. Potential participants will not be invited to join the study if the study coordinator is not able to secure an opportunity when the individual is alone long enough to adequately explain the study and obtain informed consent. By approaching the potential participant in private, she also has the opportunity to provide free consent in the absence of significant others that may affect her decision to participate. Privacy and confidentiality will further be secured by assuring that research numbers will be used in the place of personal identifiers and that no medical personnel involved in the participant's care will know whether she participates in the study.

Justice and inclusiveness are respected by including all individuals who present to the orthopaedic fracture clinic that are relevant to the research question. Age restrictions are in place in light of possible legislative requirements within the geographic areas of the recruiting clinics, and exclusion criteria have been minimized to only those individuals who have a high likelihood of providing poor quality data or being unable to properly provide free and informed consent, given that the questionnaire is available only in the primary language of each participating site (i.e. English in Canada and the United States and Dutch in the Netherlands).

Harm will be minimized by respecting the participant's privacy and affirming to her that the care she receives is in no way affected by her decision to participate or not participate in the study. In addition, Dearwater et al. noted in their study that as a safety precaution women were only administered their questionnaire while in the absence of any friends, family, or medical staff [3]. We will adopt these criteria for our study as well.

To maximize benefit, individuals who are approached to participate in the study will be offered information resources pertaining to IPV and contact information of local IPV services in the clinic area. Fracture clinic staff will also be aware of the study and will also be provided with these materials in the event that the individual approached would prefer to speak to her care provider(s) about IPV instead of members of the study team.

### Protecting Against Sources of Bias

A bias towards under-reporting IPV is our primary concern. Considerations with respect to confidentiality will be addressed during data collection to reduce bias when participants are completing the questionnaire. Participants will be approached by a female study coordinator, and the consent process and the completion of the questionnaire will take place alone in a private location so as to reduce influence from others. Additionally, participants will be told that the survey is anonymous and will be instructed to not discuss the nature of this study with other participants, so as to increase reporting of IPV, if it exists, when completing the survey (participants may answer the questionnaire differently if they knew that they would be identified as IPV victims or non-IPV victims by the individual administering the questionnaire). Questions pertaining to the participant's demographics should be subject to minimal bias because the questions are categorical and are not intended to be subjective. An under-reporting bias is certainly possible; however, if our study finds an appreciable prevalence of IPV among respondents, we will have confidence that the likely "true" estimate is even higher.

Another source of potential bias in this study is that the questionnaire is self-administered as opposed to interview administered. This may result in some missing and inconsistent data. Due to the extremely sensitive and private information, as well as taking the women's safety into consideration, we believe that an interview-administered questionnaire is not appropriate. Due to the sensitive content of the questionnaire, the study coordinator will not check it over following completion therefore she will not be able to ask the patient about missing questions or inconsistencies.

An inherent limitation of this study which may produce bias is that non-participants may differ from participants in terms of demographics and abuse prevalence. Patients may be less likely to participate if they are a victim of IPV, thus resulting in a lower IPV prevalence rate. Also, some patients may decline to participate because they are not victims of IPV and as such feel that the issue is not relevant to them. Another limitation which may lower the reported IPV prevalence is that we may miss women who are severely injured as a result of IPV because we will not approach women who are taken to the fracture clinic by Emergency Medical Services. However, we may approach these patients at subsequent follow-up visits.

### Ontario PRAISE Pilot Study

In preparation for the multi-center definitive P.R.A.I.S.E. study, we successfully completed a pilot study in Ontario, Canada at two clinical sites (Hamilton Health Sciences - General Site, Hamilton and St. Michael's Hospital, Toronto). The purpose of the pilot study was to provide estimates of patient enrollment, inform study logistics, and prove feasibility for the definitive study. The patients from the pilot study will not be included in the larger prospective study, as we have made multiple changes to the protocol and questionnaire based on our experience with the pilot study.

Our preliminary pilot work suggests that women presenting to the fracture clinics are experiencing IPV to much greater extents than previously recognized by orthopaedic surgeons. In a sample of 282 women at the two participating sites, we found that over the past 12 months 8.5% of women were physically abused, 30.5% were emotionally abused and 3.2% were sexually abused [15]. One third of women in the pilot study sample had experienced IPV (including physical, emotional and/or sexual abuse) in the past 12 months. Our pilot study also found that 2.5% of women presenting to the fracture clinics with orthopaedic injuries suffered these injuries as a direct result of IPV. The generalizability of our pilot study results to all North American, Australian, and European centers requires confirmation from our proposed larger study.

### Feasibility of the Multicenter Definitive Study

The pilot study helped to inform our enrollment rate as well as inform the study logistics. We enrolled 204 patients over five months at the site in Hamilton, which equates to approximately 40 patients per month. St. Michael's Hospital enrolled 78 patients over 2.5 months, or approximately 30 patients per month. Therefore we believe that sites will enroll between 30 and 40 patients per month and that it will take approximately 10 months of enrollment at each site to complete the pilot study. Therefore, it is feasible to complete the definitive multi-center study within the allotted timeframe.

We also made multiple revisions to the protocol and questionnaire based on our experience with the pilot study. We believe these changes will make the questionnaire easier to complete for participants and also enhance the quality of the data obtained.

### Sample Size

According to a recent survey of orthopaedic surgeons in Canada [7], 87 percent of all respondents believe that the prevalence of IPV within their practice is less than 1 percent, with almost all of the remaining respondents believing that the prevalence of IPV in their practice is between 5 percent and 10 percent. Using an estimated IPV prevalence of 5 percent within orthopaedic clinics and standard statistical formulae for estimating sample size of prevalence studies, we have calculated that a sample size of 278 women is necessary for our study to provide an estimate of IPV prevalence with a 95 percent confidence interval between 2.78 percent and 8.31 percent. If the point-estimate of IPV prevalence within orthopaedic clinics is higher than 5 percent, the proportion of the margin of error relative to the estimated prevalence will be lower. To be adequately powered within each region or demographic, each site will therefore recruit 300 participants. The total sample size for the study will be 3,600 participants across the 12 participating centers.

### Data Analysis

Data will be analyzed using SPSS Version 17.0 [SPSS, Chicago, IL] and will be stratified according to clinical center, at which point descriptive statistics can be reported for each site and overall. Dichotomous data will be reported as number of participants and proportions, with corresponding confidence intervals to estimate precision. Continuous data will be presented as means and medians with standard deviations. We will provide descriptive statistics describing the patient demographics and injury characteristics across each clinical site. We will also report the results of the WAST and PVS across each clinical site for IPV prevalence in the past 12 months. These two screening tools will be scored according to the developers guidelines. Finally, we will report descriptive statistics, with 95% confidence intervals, across each clinical site on patient's previous experiences, knowledge, and perceptions with regards to approaching health care professionals about IPV. We will use Chi-square tests (dichotomous variables) or t-tests (continuous data) to determine if there are differences in IPV prevalence across different clinical sites and jurisdictions.

### Methods Center

The Project Manager at the McMaster University Methods Center will be responsible for the overall day to day coordination of the study. The Project Manager will be responsible for communicating with the clinical sites, providing the clinical sites with the necessary case report forms and validating the data. The Project Manager will also work with the Research Coordinators at the clinical sites to ensure that the protocol is followed and that 3,600 participants are recruited for this study in the allotted timeframe.

### Participating Centers

The necessity of a multicenter study is twofold. First, as there are currently no prevalence reports of IPV in women who attend orthopaedic trauma clinics, a cross-sectional study will provide the first report of IPV prevalence in orthopaedic trauma clinics. Second, the collection of data from clinics across North America, Europe, and Australia will enhance the generalizability of our study. We will include clinical sites from a variety of trauma populations and settings (i.e. inner city versus suburban versus rural). Additional centers may be added to allow for further comparisons of the prevalence of IPV across different settings and to increase the generalizability of the findings.

### Recruitment Rate

For the multi-center definitive P.R.A.I.S.E. study we aim to recruit 3,600 participants across 12 sites in North America, Europe, and Australia. The pilot study helped to inform our enrollment rate proving feasibility of the definitive trial whereby both clinical sites in the pilot study enrolled at least 30 patients per month (St. Michael's Hospital with 30 participants per month and Hamilton Health Sciences - General Site with 40 participants per month). Consecutive patients will be recruited from fracture clinics across North America, Europe, and Australia and based on the results of our pilot study we anticipate that each site will be able to enroll 300 patients within ten months.

## Discussion

If the prevalence of IPV among women attending orthopaedic clinics is greater than the current perceptions of orthopaedic surgeons, this study will serve to advocate for the continued education of medical professionals to better recognize probable IPV cases and offer existing services to enhance the care of these patients. This is especially important because healthcare providers who receive education on screening and ways to care for IPV victims detect them more readily [27]. Furthermore, this study may encourage more open communication between orthopaedic surgeons and their patients, as two major barriers to IPV detection are either the patient is never asked [28] or the healthcare provider is reluctant to inquire [7,8,16]. A positive study will also inform the Canadian Orthopaedic Association's position statement on the role of the orthopaedic surgeon and domestic violence.

## Abbreviations

Abbreviation and Meaning: COA: Canadian Orthopaedic Association; IPV: Intimate Partner Violence; PRAISE: Prevalence of Abuse and Intimate Partner Violence Surgical Evaluation; PVS: Partner Violence Screen; WAST: Woman Abuse Screening Tool.

## Competing interests

The authors declare that they have no competing interests.

## Authors' contributions

All authors participated in conception and design of the study. All authors participated in drafting and critical revision of the manuscript. All authors read and approved the final manuscript.

## Pre-publication history

The pre-publication history for this paper can be accessed here:

http://www.biomedcentral.com/1471-2474/11/77/prepub
